# Structural and Signaling Mechanisms of Aortic Wall Failure in Heritable Thoracic Aortic Disease

**DOI:** 10.3390/cells15100936

**Published:** 2026-05-19

**Authors:** Norifumi Takeda, Hiroki Yagi, Takayuki Fujiwara, Hitomi Aono-Setoguchi, Ryo Inuzuka, Issei Komuro

**Affiliations:** 1 The Institute of Medical Science, The University of Tokyo, Tokyo 108-8639, Japan; 2 Marfan Syndrome Center, The University of Tokyo Hospital, Tokyo 113-8655, Japan; hiroki_yagi_19830414@yahoo.co.jp (H.Y.); hitomias14m@gmail.com (H.A.-S.); inuzukar-tky@g.ecc.u-tokyo.ac.jp (R.I.); 3 Department of Cardiovascular Medicine, The University of Tokyo Hospital, Tokyo 113-8655, Japan; sasasatf5804@yahoo.co.jp; 4 Department of Computational Diagnostic Radiology and Preventive Medicine, The University of Tokyo Hospital, Tokyo 113-8655, Japan; 5 Department of Pediatrics, The University of Tokyo Hospital, Tokyo 113-8655, Japan; 6 Department of Frontier Cardiovascular Science, Graduate School of Medicine, The University of Tokyo, Tokyo 113-8655, Japan; komuro_tky2000@yahoo.co.jp; 7 International University of Health and Welfare, Tokyo 107-8402, Japan

**Keywords:** heritable thoracic aortic disease, Marfan syndrome, Loeys–Dietz syndrome, transforming growth factor-β signaling, mechanotransduction, smooth muscle cell, endothelial dysfunction, extracellular matrix, aortic aneurysm and dissection, genotype–phenotype correlation

## Abstract

**Highlights:**

**What are the main findings?**

- Aortic wall failure in HTAD arises from interacting processes involving ECM structure, TGFβ signaling, and smooth muscle function.
- Disease progression reflects the combined effects of mechanical stress, endothelial responses, and immune cell involvement.

**What are the implications of the main findings?**

- Even with shared biological mechanisms, the timing and presentation of aortic events differ across genes, supporting gene-specific risk assessment.
- Linking mechanobiology with genetic information may improve clinical decision-making and the development of targeted therapies in aortic disease.

**Abstract:**

Heritable thoracic aortic diseases (HTAD) are inherited conditions that increase the risk of thoracic aortic aneurysms, dissections, and premature aortic rupture. Advances in human genetics and experimental models have transformed our understanding of these disorders from a phenotype-based classification system to a mechanism-based view involving extracellular matrix (ECM) architecture, transforming growth factor-β (TGFβ) signaling, and vascular smooth muscle cell contractility. Marfan syndrome, Loeys–Dietz syndrome, and nonsyndromic HTAD demonstrate how genetic mutations can disrupt the components that stabilize the aortic wall. These pathogenic mechanisms influence matrix organization, intracellular signaling, and the contractile machinery within the mechanically stressed proximal aorta. In this review, we summarize current mechanistic insights into the major forms of HTAD and discuss how new molecular and cellular concepts could influence surveillance, genetic counseling, and genotype-guided therapeutic strategies.

## 1. Introduction

**Figure 1:**
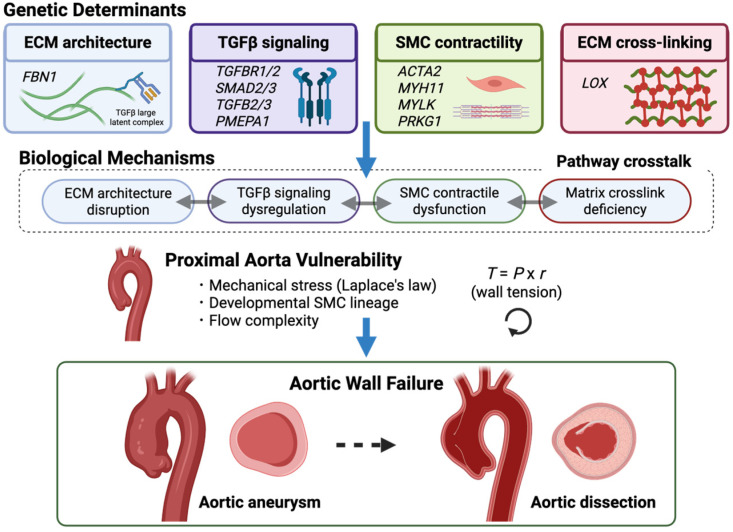
Conceptual overview of pathogenic mechanisms in HTAD; heritable thoracic aortic diseases (HTAD) arise from genetic defects that affect the extracellular matrix (ECM) architecture (e.g., *FBN1*), TGFβ signaling pathways (e.g., *TGFBR1/2*, *SMAD2/3*), smooth muscle cell (SMC) contractile machinery (e.g., *ACTA2*, *MYH11*, *MYLK*, *PRKG1*), and ECM cross-linking (e.g., *LOX*). These disturbances disrupt aortic wall homeostasis through interconnected biological mechanisms involving ECM organization, signaling dysregulation, and impaired contractile function. The proximal thoracic aorta is particularly susceptible to these disturbances due to its exposure to high mechanical stress and complex developmental architecture, which compromises its structural stability and can lead to thoracic aortic aneurysm and dissection. Created in BioRender. Norifumi Takeda. (2026). https://app.biorender.com/illustrations/6a0ad934c1ede558cd4f5e15?slideId=df52ad65-8a17-40c7-8dc8-baf4b7524ff5 (accessed on 16 April 2026).

Heritable thoracic aortic diseases (HTAD) are a group of genetic disorders that increase the risk of thoracic aortic aneurysms, dissections, and sudden aortic death. In contrast to abdominal aortic aneurysms, which are primarily degenerative and influenced by environmental risk factors, thoracic aortic disease is more often driven by inherited defects affecting the aortic wall. These conditions were historically classified based on syndromic appearance and family history. However, they are now recognized as disorders of aortic wall homeostasis, involving extracellular matrix (ECM) structure, transforming growth factor-β (TGFβ) signaling, and vascular smooth muscle cell (SMC) contractility (Figure 1). While other hereditary vasculopathies, such as vascular Ehlers–Danlos syndrome (vEDS), can also predispose to arterial rupture and dissection, vEDS more commonly affects medium- and small-sized arteries as part of a systemic vascular fragility disorder. In contrast, this review focuses on disorders characterized by predominant aortic root and proximal thoracic aorta involvement, where elastic fiber architecture, developmental smooth muscle heterogeneity, and high pulsatile biomechanical stress converge to create regional vulnerability. Emerging evidence also suggests that mitochondrial dysfunction may be another convergent mechanism contributing to aortic wall instability [1].

Studies of Marfan syndrome have demonstrated that disruption of fibrillin-1 links microfibrillar architectural defects to altered TGFβ signaling and remodeling of the aortic wall. Loeys–Dietz syndrome (LDS) further expanded this concept by showing that mutations within the TGFβ signaling pathway itself can produce severe aortopathy. In nonsyndromic HTAD (nsHTAD), most causative genes affect the smooth muscle contractile apparatus, indicating that impaired force generation represents another mechanism of aortic wall failure [2,3,4]. Collectively, these mechanisms can be broadly categorized into dysregulated TGFβ signaling and impaired smooth muscle contractility (Table 1 and Table 2).

Clinical and mechanistic features summarized from previously published studies and reviews [2,3,4,5,6]. Despite their genetic diversity, HTADs share common pathological features that compromise the structural and mechanical integrity of the thoracic aorta. Most forms predominantly affect the aortic root and proximal ascending aorta. This vascular segment is exposed to high biomechanical stress because of its large radius and direct coupling to left ventricular ejection, as predicted by Laplace’s law [3]. The proximal aorta also possesses distinctive anatomical and developmental features, including complex flow patterns and heterogeneous populations of SMCs derived from the cardiac neural crest (CNC) and the second heart field (SHF) [8,9], which may further influence the regional vulnerability of the proximal aorta in HTAD.

In this review, we discuss the mechanisms underlying Marfan syndrome, LDS, and nsHTAD, with particular emphasis on the interplay among ECM architecture, TGFβ signaling, and smooth muscle contractility within the mechanically stressed proximal thoracic aorta. We also highlight how shared pathogenic processes can result in distinct clinical phenotypes and differences in the timing and mode of aortic events across genetic backgrounds [10].

## 2. Marfan Syndrome: From Microfibrils to Mechanobiology

Marfan syndrome is caused by pathogenic variants in the *FBN1* gene, which encodes fibrillin-1, a principal structural component of extracellular microfibrils. Early mechanistic interpretations emphasized the resulting mechanical fragility due to disrupted elastic fiber architecture [11]. Microfibrils were primarily considered to be structural scaffolds that are necessary for elastogenesis and tensile resilience, and their disruption was thought to directly weaken the aortic wall.

**Figure 2:**
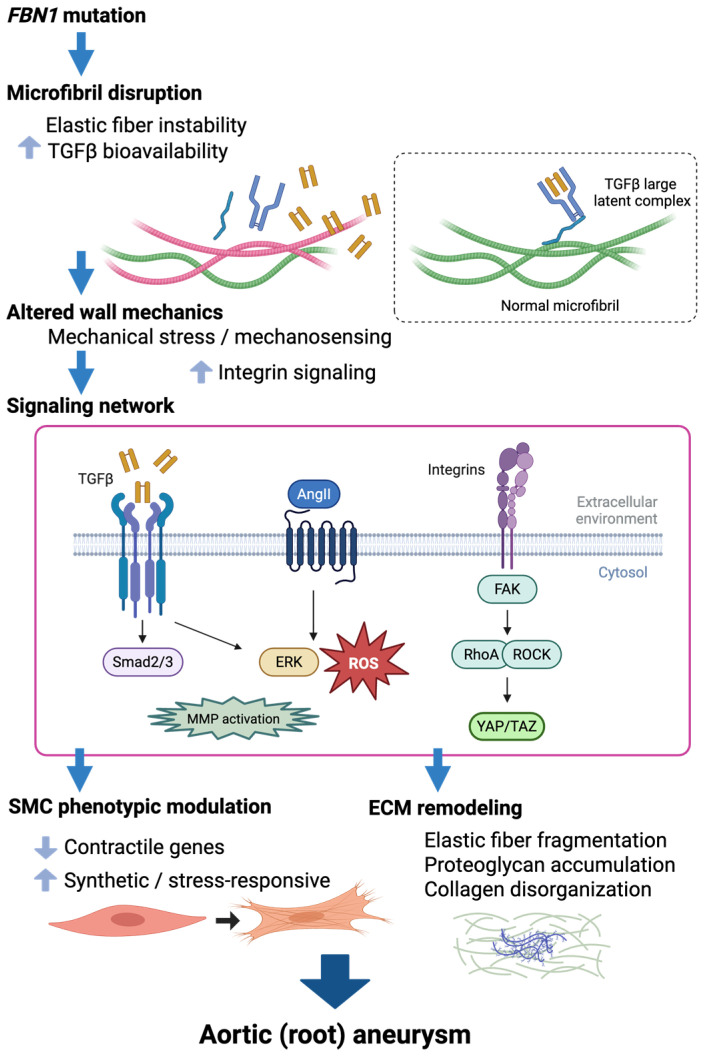
Mechanistic cascade linking *FBN1* mutations to aortic root aneurysm in Marfan syndrome; pathogenic variants in the *FBN1* gene disrupt microfibrillar architecture, resulting in elastic fiber instability and increased bioavailability of TGFβ. These changes alter aortic wall mechanics and activate mechanosensitive signaling pathways. A network of interacting cascades, including TGFβ–SMAD signaling, angiotensin II (AngII)-dependent ERK activation with associated oxidative stress, and integrin-mediated FAK–RhoA/ROCK–YAP/TAZ pathways, promotes phenotypic modulation of SMCs and ECM remodeling. These processes progressively weaken the proximal aortic wall and drive aortic root aneurysmal formation. ROS indicates reactive oxygen species; and MMP, matrix metalloproteinase. Created in BioRender. Norifumi Takeda. (2026). https://app.biorender.com/illustrations/6a0ad9bd26fca518c48e0d07?slideId=830cb269-e270-4f24-8a19-126b593cbd60 (accessed on 16 April 2026).

This structural paradigm was further refined when fibrillin-1 was shown to regulate the sequestration and activation of latent TGFβ complexes by interacting with latent TGFβ-binding proteins (LTBPs) [12]. Therefore, microfibrils perform architectural functions and act as extracellular reservoirs that spatially restrict TGFβ signaling. In *Fbn1*-mutant mice, enhanced TGFβ signaling was associated with progressive medial degeneration and aneurysm formation [13]. This led to the influential TGFβ excess hypothesis, which reframed Marfan syndrome as a disorder of dysregulated cytokine signaling. Subsequent studies have shown that both the canonical SMAD2/3 pathways and non-canonical signaling cascades contribute to disease progression. Activation of ERK was identified as a critical mediator of aneurysm development that operates independently of SMAD signaling [14]. These findings established that TGFβ signaling in Marfan syndrome is multifaceted and stage-dependent, involving parallel intracellular pathways with distinct biological consequences (Figure 2).

Recent studies show that pathogenic *FBN1* variants alter the mechanical properties of the ECM [15,16]. Changes in matrix compliance and microfibrillar organization affect how cells transmit force, influencing integrin engagement and downstream FAK signaling. These changes also intersect with the RhoA/ROCK and YAP/TAZ pathways [17,18,19,20]. In this context, abnormal mechanical stress modulates and amplifies cytokine signaling rather than replacing it, linking extracellular architecture to intracellular signal diversification. Recent studies also suggest that ECM remodeling itself can actively amplify mechanotransductive signaling. Specifically, increased fibronectin deposition in the medial layer enhances integrin α5-dependent signaling, promoting inflammatory activation and SMC phenotypic modulation in Marfan aortopathy [21]. The fibronectin–integrin axis therefore represents an additional mechanism by which ECM remodeling can lead to changes in intracellular signaling during aneurysm progression.

Accordingly, the conceptual understanding of Marfan syndrome has evolved from a model based solely on structural defects, to a signaling-centered paradigm, and finally to an integrated perspective. The interaction between ECM architecture, TGFβ regulation, and mechanotransduction influences cellular fate within the aortic wall [22,23,24].

### 2.1. FBN1 Genotype and Variant-Specific Mechanisms in Marfan Syndrome

The *FBN1* gene is located on chromosome 15q21.1 and encodes fibrillin-1, a large ECM glycoprotein composed of multiple calcium-binding epidermal growth factor-like (cbEGF) domains and TGFβ binding protein-like (TB) motifs [25]. These domains form an elongated protein that can assemble into extracellular microfibrillar networks, providing structural support to elastic tissues. Microfibrils also participate in elastogenesis and regulate extracellular signaling by interacting with latent TGFβ complexes [26,27].

More than 4000 pathogenic *FBN1* variants have been identified in patients with Marfan syndrome [28]. These variants include missense, truncating, splice-site, and small insertion or deletion mutations, as well as larger deletions that affect the entire gene or a significant portion of it [29]. Since fibrillin-1 is a large, multidomain protein involved in complex extracellular assembly processes, different classes of variants can disrupt its biology through distinct molecular mechanisms. This leads to significant heterogeneity in microfibril architecture and downstream cellular responses. Two mechanisms have been proposed to explain these variant-specific effects: haploinsufficiency and the dominant-negative disruption of microfibrils. Haploinsufficiency is typically caused by variants that introduce premature termination codons (PTCs). In most cases, transcripts harboring PTCs are recognized by the nonsense-mediated mRNA decay (NMD) surveillance pathway and undergo degradation [30]. This mechanism is sometimes referred to as functional haploinsufficiency because fibrillin-1 production is reduced through degradation of mutant transcripts. In addition, true haploinsufficiency resulting from complete deletion or lack of expression of one *FBN1* allele has also been shown to be sufficient to cause Marfan syndrome [31,32]. Decreased fibrillin-1 availability in this scenario leads to reduced microfibrillar density within the ECM. In contrast, dominant-negative variants, which are often missense mutations affecting cysteine residues within cbEGF domains, can allow mutant fibrillin-1 proteins to be incorporated into assembling microfibrils. This disrupts the structure and stability of microfibrils.

Clinical cohort studies suggest that these variant classes may influence disease severity differently. Several analyses indicate that haploinsufficient variants are associated with faster aortic dilation and earlier aortic events than dominant-negative variants [33,34] (Table 1). One possible explanation is that reduced microfibril abundance compromises the mechanical buffering capacity of the aortic wall, thereby increasing susceptibility to hemodynamic stress. However, genotype–phenotype correlations in Marfan syndrome are not absolute. Even among family members with identical *FBN1* variants, the rate of aortic enlargement and the timing of complications can differ significantly. These observations suggest that additional modifiers, including genetic background, environmental influences, and biomechanical factors, likely contribute to the ultimate clinical trajectory.

Experimental models have also provided insights into genotype-dependent mechanisms, although important limitations exist. Much of the mechanistic work on Marfan aortopathy relies on two widely used mouse models. The first is the *Fbn1*^mgR/mgR^ hypomorphic mouse model [35], which expresses markedly reduced levels of fibrillin-1. The second is the *Fbn1*^C1041G/+^ knock-in mice [36,37], which represents a missense *FBN1* variant that affects microfibril assembly. The hypomorphic *Fbn1*^mgR/mgR^ mice exhibit rapid and severe aortic disease progression, which is consistent with the idea that reduced levels of fibrillin-1 can severely destabilize the mechanics of the aortic wall. However, the limited number of models is an important experimental constraint, considering the extensive diversity of human *FBN1* variants and the wide spectrum of clinical phenotypes observed in patients.

The genotype–phenotype relationships associated with Marfan syndrome extend beyond the aorta and affect multiple organ systems, where microfibrils play a role in maintaining the integrity of connective tissue [38,39]. Certain *FBN1* variants have been associated with severe, early-onset presentations, such as neonatal Marfan syndrome, in which pathogenic variants frequently cluster within the so-called neonatal region spanning exons 25–33 [40]. For ocular manifestations, clustering of variants associated with ectopia lentis has been reported within the N-terminal region of *FBN1*, particularly involving cysteine substitutions in exons 1–15 and the broader exon 1–20 region [41]. Skeletal manifestations also exhibit genotype-associated variability. The progression of scoliosis, a major clinical management issue during adolescence, differs according to *FBN1* variant classes [42]. Cardiac valvular disease is another example of genotype-dependent disease expression. Mitral valve prolapse and regurgitation are common cardiovascular conditions that require surgical intervention for some patients. Recent genotype-stratified analyses have shown that the molecular class and genomic location of *FBN1* variants influence the timing and risk of mitral valve surgery [43]. Specifically, in-frame variants within DNCD regions spanning exons 26–37 and 44–50 were associated with a markedly higher cumulative incidence of mitral valve surgery than other in-frame variants or premature termination codon variants. Notably, these DNCD variants were associated with earlier onset of mitral valve disease, frequently during childhood or adolescence, whereas individuals with truncating variants exhibited a delayed risk profile that increased later in adulthood.

Overall, the *FBN1* genotype influences organ-specific patterns of disease susceptibility, affecting not only aortic pathology, but also valvular and systemic manifestations. Although genotype alone cannot fully predict individual outcomes and the mechanisms underlying these genotype–phenotype relationships are unclear, genetic information remains an essential component of lifelong clinical surveillance for Marfan syndrome. Looking to the future, a deeper understanding of genotype-specific mechanisms could inform the development of precision medicine approaches. Since *FBN1* variants affect the structure of the ECM, the regulation of TGFβ, and mechanotransduction pathways, it is possible that different variant classes may respond differently to pharmacological interventions targeting these pathways. Interindividual genetic variability may also influence therapeutic responsiveness. Such genotype-specific therapeutic strategies are still being investigated, but combining molecular genetics with mechanistic insight could improve risk stratification and the management of patients with Marfan syndrome. From a clinical perspective, these genotype–phenotype correlations already inform discussions about surgical decision-making, because haploinsufficient variants have been associated with faster aortic growth and earlier aortic events than dominant-negative variants. In selected patients, this may justify closer surveillance and consideration of prophylactic surgery toward the lower end of current guideline thresholds. In large cohort studies, *FBN1* variant class has been linked to differences in the timing of aortic complications as well as extra-aortic interventions, highlighting the importance of integrating molecular diagnosis into individualized risk assessment and counseling for Marfan syndrome [28,33,38,43].

### 2.2. TGFβ Signaling as a Central but Context-Dependent Mechanism in Marfan Syndrome

Recognition of the fact that fibrillin-1 regulates the sequestration and activation of latent TGFβ complexes established that dysregulated TGFβ signaling is a central mechanism of Marfan syndrome [13,36,44]. Initially, canonical signaling through SMAD2/3 phosphorylation was considered the primary pathogenic pathway. This idea was supported by the observation of increased nuclear SMAD2 phosphorylation observed in aneurysmal aortic tissue and by the attenuation of aortic dilation in murine models following pharmacological modulation of TGFβ signaling. These findings reinforced the concept that excessive TGFβ activity functions as a primary driver of the disease.

However, subsequent work has significantly refined this concept. Temporal analyses in *Fbn1*-mutant mice revealed that TGFβ signaling is not uniformly harmful, but rather exhibits stage-dependent effects. For example, suppressing TGFβ signaling during the early stages of postnatal development can worsen aortic pathology [45,46], suggesting that basal TGFβ activity participates in adaptive or compensatory remodeling during this period. In contrast, sustained or dysregulated signaling later in disease progression contributes to progressive medial degeneration [47]. These observations challenge the linear “more TGFβ equals worse disease” paradigm, suggesting that disease progression likely reflects a context-dependent imbalance in pathway activation.

Crucially, it became evident that TGFβ receptor engagement activates not only canonical SMAD2/3 signaling, but also non-canonical cascades [14]. Sustained activation of ERK was identified as a critical mediator of aneurysm development that can drive pathological remodeling independently of SMAD phosphorylation. In *Fbn1*^C1041G/+^ mice, the pharmacological inhibition of ERK signaling reduced aortic root dilation despite persistent SMAD2 activation. This finding suggests that the MAPK pathway has an autonomous pathogenic potential and expands the mechanistic landscape beyond SMAD-centered signaling. Mechanistically, ERK activation in Marfan aortopathy is closely linked to angiotensin II (AngII) signaling through the AT1 receptor (AT1R) [13,14,48]. Enhanced AT1R stimulation promotes ERK phosphorylation, increases matrix metalloproteinase (MMP) expression, and amplifies SMC phenotypic modulation. Losartan, an AngII type 1 receptor blocker (ARB), has been shown to reduce ERK activation and prevent aortic dilation in *Fbn1^C1041G/+^* mice, positioning the renin–angiotensin system as a functional bridge between ECM disruption and intracellular stress signaling. Notably, the protective effect of losartan in murine models depends on AngII type 2 receptor (AT2R) signaling; genetic deletion of *At2r* abolishes losartan’s ability to suppress ERK activation and aneurysm progression [14]. Recent work further suggests that ARBs exert protective effects not only through competitive inhibition of AngII–AT1R signaling but also via inverse agonism at the AT1R, which suppresses basal receptor activity and modulates downstream ERK signaling and endothelial adaptive responses [49]. However, emerging evidence indicates that suppression of aneurysm growth does not necessarily normalize the mechanical integrity of the aortic wall. For instance, losartan treatment reduced aneurysm formation in *Fbn1*^mgR/mgR^ mice, but did not improve aortic rupture force, indicating persistent biomechanical vulnerability despite attenuated aortic dilation [15].

In addition to ERK, other non-canonical pathways, including p38 MAPK and JNK, influence apoptosis, inflammatory gene expression, and ECM remodeling programs in vascular SMCs [45,50]. PI3K/Akt signaling has also been reported to intersect with TGFβ receptor activation in various cellular contexts [51,52]. Overall, these data demonstrate that TGFβ receptor signaling in Marfan syndrome operates as an interconnected network of intracellular cascades, the relative contributions of which vary according to developmental stage, biomechanical stress, and cellular context.

These observations provided the rationale for evaluating ARBs as a disease-modifying therapy. Although trial results have shown variability, ARBs have demonstrated at least non-inferiority to β-blockade in limiting aortic root enlargement in several cohorts, supporting the translational relevance of AngII–ERK modulation [53,54,55]. Pharmacogenetic factors may partly contribute to variability in treatment response. Since losartan requires CYP2C9-dependent conversion to the active metabolite E-3174, patients carrying reduced-function CYP2C9 alleles may exhibit altered therapeutic responsiveness, potentially warranting consideration of pharmacogenetic information in treatment individualization [56]. However, incomplete disease suppression indicates that targeting a single signaling axis is unlikely to fully normalize the complex, context-dependent signaling environment present in Marfan aortopathy.

### 2.3. Endothelial Cell Responses Under Abnormal Mechanical and Oxidative Stress

ECs are uniquely positioned at the luminal interface to sense the altered shear stress and cyclic strain generated by disrupted aortic biomechanics. In Marfan syndrome, pathogenic *FBN1* variants disrupt microfibrillar architecture and elastic recoil. These changes modify pulse wave propagation and the distribution of regional shear stress along the aortic wall [35,57]. Although TGFβ dysregulation is central to the disease’s pathobiology, these hemodynamic changes introduce an additional layer of signaling complexity operating at the endothelial surface.

ECs are highly mechanosensitive. Under physiological laminar flow conditions, transcription factors such as KLF2 and KLF4 promote nitric oxide (NO) production and anti-inflammatory homeostasis [58]. In contrast, disturbed flow and increased cyclic stretch enhance the activation of NADPH oxidase and the generation of mitochondrial reactive oxygen species [59]. In *Fbn1*^C1041G/+^ mice, increased oxidative stress within the aortic wall has been documented, with contributions from the NADPH oxidase and xanthine oxidoreductase pathways [20,60]. Oxidative stress directly intersects with TGFβ signaling, and reactive oxygen species can activate latent TGFβ complexes and enhance SMAD and ERK pathway activity downstream [61]. Conversely, TGFβ signaling induces pro-oxidant enzymes such as NADPH oxidase 4 (NOX4), thereby reinforcing the interaction between cytokine signaling and oxidative stress [62]. These pathways interact closely rather than acting independently.

In this context, endothelial dysfunction encompasses more than just reduced NO bioavailability. ECs that are mechanically and oxidatively stressed secrete paracrine mediators that influence medial remodeling. These mediators include [63] (i) CTGF (CCN2), a downstream effector of TGFβ signaling that promotes ECM remodeling, (ii) PDGF-B, which enhances SMC migration and phenotypic modulation, (iii) endothelin-1 [64], which contributes to vasoconstriction and medial stress amplification, and (iv) IL-6 and MCP-1 (CCL2), which facilitate inflammatory cell recruitment and secondary matrix degradation. Through these mediators, ECs translate altered mechanical and cytokine cues into coordinated SMC and inflammatory responses.

Recent single-cell RNA sequencing studies have further demonstrated that the aortic endothelium is transcriptionally heterogeneous [65,66]. Different endothelial subpopulations exhibit varying levels of mechanosensitive genes, inflammatory programs, and TGFβ-responsive signatures in Marfan aortopathy. Subpopulations characterized by transcriptional profiles associated with oxidative stress appear to increase during aneurysmal formation or dissection. This suggests that disease evolution may preferentially involve specific endothelial states rather than a simple, uniform endothelial dysfunction.

Collectively, these findings support a model in which ECs actively integrate TGFβ signaling, mechanical stress, and oxidative pathways. Rather than serving as passive barriers, ECs contribute to medial remodeling by activating oxidative stress-dependent signaling and paracrine mechanisms that interact with canonical and non-canonical TGFβ cascades. Although endothelial responses have traditionally been considered to modulate disease progression, recent experimental evidence indicates that endothelial dysfunction may also contribute to the initiation of aortic wall failure. In a newly developed *Fbn1*^G234D/G234D^ mutant model, impaired endothelial mechanosensing and intimal macrophage accumulation occurred prior to the formation of intimomedial tears under conditions of reduced TGFβ signaling, highlighting the potential initiating role of endothelial–immune interactions [67].

### 2.4. Smooth Muscle Cell Heterogeneity and Signal Integration

Although endothelial signaling integrates luminal mechanical and cytokine cues, the medial SMC layer remains the primary structural determinant of aortic wall integrity in Marfan syndrome. Medial degeneration has been described as “cystic medial degeneration” or medial necrosis. It is characterized by SMC loss, elastic fiber fragmentation, and accumulation of proteoglycan-rich ECM [68]. Within this pathological context, SMCs were largely viewed as passive victims of ECM disorganization and structural weakening. However, accumulating experimental and human data now indicate that SMCs actively interpret and amplify matrix-derived and cytokine-mediated signals, thereby contributing directly to aneurysmal progression.

TGFβ signaling exerts central regulatory control over SMC phenotype. Canonical SMAD2/3 activation is essential for regulating the expression of contractile genes (e.g., *ACTA2*, *MYH11*, and *TAGLN*) and ECM components (e.g., *COL1A1* and *ELN*) [69,70]. Increased nuclear SMAD2 phosphorylation has been consistently observed in medial SMCs in *Fbn1*^C1041G/+^ mice [13]. Non-canonical pathways, including ERK [45], p38 MAPK [50] and JNK [45], and PI3K/Akt [71,72], also intersect with TGFβ receptor activation and modulate proliferation, apoptosis, metabolic adaptation, and inflammatory gene expression. These cascades function as an integrated signaling network rather than a linear axis. They produce diverse cellular outputs depending on mechanical and oxidative conditions. However, as noted above, TGFβ signaling is stage-dependent and context-sensitive.

SMCs are intrinsically mechanosensitive. Integrin-mediated adhesion complexes and FAK signaling translate ECM stiffness and tensile load into intracellular responses. Then, RhoA/ROCK signaling regulates actin polymerization and the stability of the contractile apparatus. Meanwhile, YAP/TAZ transcriptional coactivators respond to cytoskeletal tension and mechanical strain [17,18,19,20]. Altered matrix compliance and progressive dilation in Marfan aortopathy disrupt circumferential stress distribution, leading to dysregulated cytoskeletal tension. Reduced expression of contractile markers (*ACTA2* and *MYH11*) and increased expression of synthetic markers (*COL1A1* and *FN1*) reflect a shift toward a modulated phenotype [21,73,74]. This shift represents a continuum of transcriptional states influenced by mechanical load, TGFβ signaling, and intracellular stress responses, rather than a binary switch.

Oxidative stress also modulates SMC fate. Increased ROS production has been documented in Marfan models, including vascular SMCs, and is associated with enhanced ERK activation and MMP-dependent ECM degradation [75,76,77]. NOX4 expression is markedly increased in vascular SMCs, resulting in redox stress that targets contractile cytoskeletal proteins, promoting elastic fiber fragmentation, aortic root dilation, and endothelial dysfunction. Genetic deletion of *Nox4* improves these medial abnormalities in *Fbn1*^C1041G/+^ mice [78]. Oxidative stress is also associated with increased MMP2 and MMP9 activity, which promotes elastic fiber fragmentation and medial weakening [79]. Thus, oxidative pathways interact with canonical and non-canonical TGFβ cascades to amplify structural deterioration.

Another line of research has examined the role of proteoglycan-rich ECM remodeling in disease progression. Versican, a large chondroitin sulfate proteoglycan enriched in regions of medial degeneration, modulates SMC signaling. Recent experimental studies have demonstrated that versican accumulation activates Akt-dependent pathways and induces Nos2-related NO signaling. This promotes inflammatory activation and ECM remodeling within the aortic wall [72]. These observations suggest that proteoglycan accumulation, long considered a histopathological hallmark of medial degeneration, may actually function as an active driver of cellular stress responses rather than merely being a secondary structural change [80].

Single-cell transcriptomic studies have fundamentally altered our understanding of the role of SMCs in Marfan aortopathy [21,65]. Rather than being a uniform contractile population, medial SMCs exist along multiple transcriptional trajectories. Recent studies using lineage tracing and single-cell transcriptomic approaches have demonstrated that vascular SMCs exhibit substantial phenotypic plasticity during aneurysmal formation. They transition from contractile states towards matrix-remodeling, inflammatory or stress-responsive phenotypes. This suggests that SMC heterogeneity is a dynamic spectrum of cellular states rather than a binary contractile–synthetic switch [81]. A disease-associated cluster of phenotypically modulated SMCs (“modSMCs”) was identified in *Fbn1*^C1041G/+^ mice and human Marfan aortic root tissue. These cells exhibit (i) downregulation of contractile genes (*ACTA2*, *MYH11*, *TAGLN*), (ii) upregulation of matrix-remodeling genes (*COL1A1*, *FN1*, *VCAN*, *LUM*, *DCN*), (iii) increased expression of KLF4, consistent with KLF4-dependent modulation programs described in atherosclerosis [82], and (iv) enrichment of stress- and injury-response signatures.

In the dissecting *Fbn1*^mgR/mgR^ model, two discrete aortic cell subpopulations (SMC3 and EC4) were identified and combined into a disease-specific state, “MFSmod.” SMC3 is enriched in ECM- and NO signaling-related genes, and the MFSmod state is specifically associated with dissecting thoracic aortic aneurysm in mouse modeling Marfan syndrome and an increased risk of aortic dissection in patients with Marfan syndrome [83]. Trajectory analyses predict directional transitions between contractile SMCs and the modulated SMC3/EC4 states in the dissecting *Fbn1*^mgR/mgR^ model, indicating a distinct, dynamically regulated cell population exclusively associated with dissecting thoracic aortic aneurysm. The emergence of these modulated populations in response to losartan treatment suggests that SMC state plasticity remains pharmacologically modifiable.

### 2.5. Inflammatory Cell Involvement and Immune Amplification

Beyond endothelial and SMC–intrinsic signaling, inflammatory cells have emerged as active modulators in Marfan aortopathy. Histological analyses have long demonstrated the accumulation of macrophages within aneurysmal tissue [84]. Notably, infiltration of inflammatory cells is often more prevalent in the adventitia, suggesting that inflammation in the outer aortic wall may contribute to medial remodeling.

In both human Marfan specimens and murine models, macrophages are also enriched in the adventitia [85,86]. This spatial distribution may be mechanistically relevant because adventitial immune activation can affect medial SMCs through cytokine diffusion, matrix-remodeling enzymes, and vasa vasorum-associated signaling. A recent study demonstrated that modulating TGFβ signaling in the myeloid lineage significantly alters aneurysm progression in *Fbn1*^C1041G/+^ mice [85]. The study found that altering TGFβ receptor signaling in myeloid cells influenced SMC proliferation and vascular remodeling. These results suggest that immune cells actively shape the local signaling environment in response to matrix damage. This finding supports the idea that the adventitia functions as an active immunological niche rather than a passive structural layer in aortic aneurysm disease [87,88].

Mechanistically, macrophages can activate latent TGFβ complexes through integrin-dependent mechanisms, such as αvβ8-mediated activation [89]. TGFβ signaling, meanwhile, regulates macrophage polarization and cytokine production, promoting an M2-like phenotype with altered pro- and anti-inflammatory cytokine profiles [90]. In aortic tissue predisposed to dysregulated TGFβ availability due to *FBN1* mutations, immune cell-dependent modulation may further skew the local TGFβ signaling balance. Oxidative stress enhances inflammatory activation through NF-κB-dependent transcriptional programs. Macrophage-derived reactive oxygen species and proteases, such as MMP9, contribute to elastin degradation and ECM destabilization within the aneurysmal aortic wall [77]. Oxidative stress can potentiate TGFβ signaling by inducing TGFβ expression and promoting the activation of latent TGFβ complexes, thereby creating a feed-forward interaction among immune activation, cytokine signaling, and structural weakening [91,92].

Recent single-cell analyses have revealed substantial heterogeneity within infiltrating immune populations in aortic aneurysms [93,94,95]. Distinct macrophage subsets enriched for chemokine signaling, matrix-degrading enzymes, or angiogenic mediators have been identified in aneurysmal tissue. Histological and imaging studies further indicate that immune infiltrates often concentrate in the adventitia and peri-medial regions. This suggests that the diversity of immune cells may influence the spatial pattern and tempo of aortic wall remodeling.

Collectively, inflammatory cells in Marfan syndrome appear to act as signal amplifiers and modulators within a vascular wall environment that is already primed by mechanical stress and dysregulated TGFβ signaling. The abundance of adventitial macrophages highlights the fact that disease progression cannot be conceptualized as solely luminal or medial pathology. Rather, remodeling reflects dynamic, bidirectional communication across the endothelial, medial, and adventitial compartments. Thus, the adventitia can function as an active immunological niche in aortic disease, promoting inflammatory responses within the vascular wall.

### 2.6. Integrated Mechanisms of Marfan Aortopathy

Heritable aortopathies result from the failure of a mechanically specialized vascular segment rather than from general arterial weakness. The proximal thoracic aorta is exposed to unique pulsatile loading, complex flow patterns, and developmental heterogeneity of SMC lineages. In this environment, alterations in ECM architecture, TGFβ signaling, smooth muscle contractility, oxidative stress, and immune modulation interact to destabilize aortic wall homeostasis. This helps explain the proximal aorta’s regional vulnerability and the heterogeneous clinical progression observed in HTAD (Figure 1 and Figure 2).

## 3. Loeys–Dietz Syndrome: Receptor Mutations and the TGFβ Signaling Paradox

### 3.1. Clinical Characteristics and Phenotypic Spectrum

LDS is an HTAD characterized by aggressive arterial aneurysm formation and a broad spectrum of systemic connective tissue manifestations [96,97]. Originally, LDS was clinically defined by a triad consisting of arterial tortuosity, hypertelorism, and a bifid uvula or cleft palate. These features distinguish LDS from other heritable aortopathies [98,99,100]. However, the phenotypic spectrum of LDS is remarkably heterogeneous. Some patients exhibit craniofacial and skeletal manifestations resembling those observed in Marfan syndrome, whereas others present with relatively subtle systemic findings and a nearly normal body habitus, despite having significant vascular disease.

### 3.2. Genetic Basis and Genotype–Phenotype Correlations

Genetic studies have established LDS as a disorder involving dysregulated TGFβ signaling, which is caused by pathogenic variants affecting multiple levels of the TGFβ signaling cascade. These variants include receptor genes, such as *TGFBR1* and *TGFBR2* [98,99], intracellular mediators, such as *SMAD3* [101,102] and *SMAD2* [103], and the ligands *TGFB2* [46,104] and *TGFB3* [105,106,107]. Variants in *SMAD3* were initially identified in patients with aneurysm-osteoarthritis syndrome, which is characterized by thoracic aortic aneurysm accompanied by early-onset osteoarthritis and arterial tortuosity, suggesting the pleiotropic consequences of disrupted canonical TGFβ signaling [101]. More recently, variants in *PMEPA1*, a negative regulator of TGFβ signaling that modulates SMAD-dependent transcriptional feedback, have been identified as an additional genetic cause of LDS-like aortopathy [108]. Notably, genotype-specific differences in vascular severity have been observed within LDS. Variants in *TGFBR1*, *TGFBR2*, and *SMAD3* are frequently associated with earlier and more aggressive arterial manifestations compared with other LDS-associated genes [109,110], indicating the importance of pathway position and signaling context in determining vascular phenotype (Table 1).

More detailed clinical analyses have revealed significant differences even between the two receptor genes. For example, sex-related differences in vascular severity seem to depend on the affected gene. Patients with *TGFBR1* variants exhibit significant sex-based differences; males experience aortic events earlier and more frequently. In contrast, such sex differences are largely absent among individuals with *TGFBR2* variants [109]. However, *TGFBR2* risk is not uniform. Current guidelines recognize smaller body size, particularly in women, as well as extra-aortic features, family history of dissection, and rapid aortic growth (≥0.3 cm/year) as high-risk features [5]. Additionally, several systemic features have been identified as clinical predictors of vascular risk in LDS. Genotype-stratified analyses have associated the presence of hypertelorism, pronounced arterial tortuosity, and translucent skin with an increased likelihood of aortic events [109].

### 3.3. Systemic Arteriopathy and Vascular Phenotype

Arterial tortuosity is a characteristic vascular feature of LDS that likely reflects alterations in vascular wall development driven by dysregulated TGFβ signaling [111]. TGFβ pathways play a key role in vascular morphogenesis, SMC differentiation, and ECM organization during arterial development [112,113]. Disruption to these processes can result in progressive arterial elongation in response to pulsatile hemodynamic forces, producing the characteristic tortuous vascular phenotype observed in LDS [100,114,115].

Consistent with this mechanism, vascular involvement in LDS extends well beyond the proximal aorta. Aneurysms and dissections frequently occur throughout the arterial tree, including the carotid, vertebral, subclavian, mesenteric, and intracranial arteries. Head and neck vessels, in particular, often demonstrate marked tortuosity and aneurysmal remodeling. The prominent involvement of the head and neck arteries may reflect the fact that neural crest-derived vascular structures depend on tightly regulated TGFβ signaling during embryonic vascular development [111]. Furthermore, the degree of arterial tortuosity has been suggested as an indicator of LDS disease severity and vascular fragility [109], reflecting systemic disturbance to the vascular wall structure.

Another important clinical feature of LDS is that aortic dissections tend to occur at smaller aortic diameters than those seen in other heritable aortopathies. Clinical observations suggest that fatal aortic events can occur even when the aortic root is only moderately enlarged. Consequently, the threshold for structural failure may be reached at smaller vessel diameters. These observations have important clinical implications and have led to recommendations for more careful, longitudinal surveillance and earlier surgical intervention for patients with LDS than for those with other forms of HTAD.

### 3.4. TGFβ Signaling Dysregulation and the Paradox of Receptor Mutation

**Figure 3:**
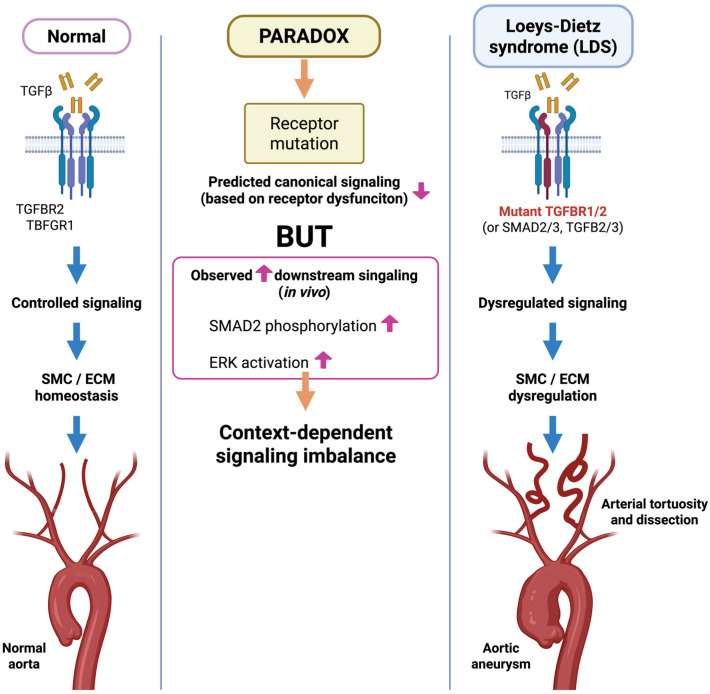
Paradoxical activation of TGFβ signaling in Loeys–Dietz syndrome; in normal aortic tissue, TGFβ receptor signaling is tightly regulated to maintain SMC and ECM homeostasis. In Loeys–Dietz syndrome (LDS), pathogenic variants affecting TGFβ receptors or related pathway components are predicted to impair canonical signaling. However, in vivo observations demonstrate enhanced downstream signaling, including increased SMAD2 phosphorylation and ERK activation. This paradoxical imbalance in signaling drives SMC and ECM dysregulation, resulting in arterial tortuosity, aneurysm formation, and dissection. Created in BioRender. Norifumi Takeda. (2026). https://app.biorender.com/illustrations/6a0ad9fba5fb99e48accd41b?slideId=5d7187a3-4f4c-4593-b0c9-fa292dd2c094 (accessed on 16 April 2026).

The molecular pathogenesis of LDS presents a distinctive paradox in vascular biology [116]. LDS is caused by heterozygous mutations in genes that encode components of the TGFβ signaling pathway, most notably the TGFBR1 and TGFBR2 receptors. In principle, these variants would be expected to reduce receptor-mediated signaling. However, affected aortic tissues consistently demonstrate increased downstream signaling activity, including enhanced phosphorylation of SMAD2 and activation of MAPK pathways, particularly ERK [99,100,114]. This apparent contradiction, often referred to as the “TGFβ paradox,” remains one of the defining conceptual features of LDS pathobiology [117,118,119]. Importantly, this paradox does not imply that receptor mutations directly increase kinase activity. Rather, many LDS-associated variants appear to impair or qualitatively alter canonical receptor-mediated signaling, while promoting context-dependent hyperactivation of the kinase in vivo. Experimental evidence indicates that impaired receptor function can disrupt negative feedback regulation, alter ligand availability, and increase susceptibility to secondary signaling inputs, including AngII-dependent ERK activation. Consequently, the net effect within the aortic wall is the paradoxical activation of downstream signaling, despite the presence of mutations that are predicted to reduce receptor function. Furthermore, the magnitude and direction of these signaling abnormalities are highly context-dependent, varying with cell lineage, biomechanical environment, and disease stage. Together, these findings suggest that LDS is not merely a disorder of reduced TGFβ signaling but rather a disease of dysregulated signaling homeostasis. Studies of LDS patient tissue and experimental models therefore suggest that receptor variants impair canonical receptor signaling while promoting the dysregulated activation of downstream signaling pathways (Figure 3).

Studies have provided insight into the underlying mechanisms of this paradox by demonstrating that AngII signaling through the AT1R can amplify TGFβ pathway activity in LDS. Experimental work in LDS knock-in mouse models has shown that AngII-dependent stimulation of downstream pathways, including ERK, can promote aneurysmal formation despite impaired receptor function. This establishes a link between neurohormonal signaling and dysregulated TGFβ signaling networks [114], and suggests that LDS is not merely a disorder of reduced receptor activity, but rather a disease involving context-dependent signaling imbalance. Mutations affecting TGFβ receptors can disrupt feedback regulation, resulting in the excessive activation of downstream signaling pathways within the aortic wall.

Further support for the importance of regulatory control within the TGFβ pathway comes from the recent identification of *PMEPA1* as a genetic cause of LDS-like aortopathy [108]. *PMEPA1* encodes a TGFβ-inducible negative regulator of SMAD signaling that functions as part of a feedback mechanism that limits pathway activation [120]. Therefore, pathogenic variants in *PMEPA1* are predicted to impair this feedback control, permitting excessive downstream signaling despite structurally intact TGFβ receptors. While this mechanism is specific to *PMEPA1*-associated aortopathy, it demonstrates how the disruption of TGFβ signaling homeostasis, rather than the mere loss of receptor function, can result in vascular phenotypes resembling LDS.

The SMC lineage appears to play a critical role in determining the regional susceptibility to aneurysmal formation in LDS [121,122,123]. In the proximal thoracic aorta, medial SMCs originate from two distinct embryonic sources: CNC and SHF. Experimental lineage-specific disruption of TGFβ receptor signaling demonstrated that these populations respond differently to altered TGFβ signaling. Deletion of *Tgfbr2* in SHF-derived SMCs resulted in pronounced medial degeneration, elastin fragmentation, and progressive dilation of the proximal aorta. In contrast, disruption of signaling in CNC-derived cells produced substantially milder vascular changes. SHF-derived SMCs also exhibit enhanced sensitivity to AngII-dependent signaling, which converges on ERK and related pathways associated with vascular remodeling [121]. Because SHF-derived cells are enriched in the proximal ascending aorta and the aortic root, this lineage-specific hypersensitivity to AngII signaling may contribute to the regional vulnerability of the proximal aorta and may also help explain the paradoxical increase in downstream signaling activity observed in LDS despite receptor mutations. Therefore, these observations indicate that the vascular consequences of altered TGFβ signaling are strongly influenced by developmental cell lineage and local signaling context rather than by receptor dysfunction alone. This is consistent with the concept of regional vulnerability within the aorta in which developmental origin, local biomechanical forces, and signaling networks interact to determine site-specific susceptibility to aneurysm formation.

Collectively, these observations indicate that LDS represents a prototypical signaling-driven aortopathy in which disruption of the TGFβ pathway perturbs vascular development, SMC homeostasis, and the biomechanical integrity of the arterial wall.

## 4. Nonsyndromic Heritable Thoracic Aortic Disease

nsHTAD is a condition that encompasses familial thoracic aortic aneurysm and dissection occurring in the absence of the systemic features typical of syndromic connective tissue disorders, such as Marfan syndrome or LDS [4,25,124]. Many affected individuals appear clinically unremarkable or have only mild, nonspecific physical findings that are insufficient to suggest a defined genetic syndrome. Nevertheless, familial clustering of thoracic aortic aneurysms and dissections is common. Approximately 20% of patients with thoracic aortic disease report a positive family history, supporting the substantial inherited contribution to disease susceptibility.

Despite major advances in genomic technologies, the genetic architecture of nsHTAD remains incompletely defined. Even among families with a strong history of disease, established causative genes, including *ACTA2*, *MYH11*, *MYLK*, *PRKG1*, and *LOX*, explain only a minority of cases, and pathogenic or likely pathogenic variants are typically identified in only 10–20% of nsHTAD patients. The diagnostic yield varies across cohorts and testing strategies. This limited diagnostic yield highlights the significant genetic heterogeneity of nsHTAD and suggests the involvement of additional rare variants, such as structural and regulatory changes, as well as oligogenic or polygenic contributions, along with other unrecognized mechanisms.

Genome-wide association studies (GWAS) have identified several common variants associated with aortic diameter and susceptibility to thoracic aortic disease. However, these loci generally have modest effect sizes and low penetrance; therefore, they are considered susceptibility modifiers rather than primary determinants of aortic pathology [4]. The strong familial aggregation observed in many nsHTAD families contrasts with the relatively small proportion of cases explained by known high-impact variants and common risk alleles. This suggests substantial “missing heritability,” likely reflecting undiscovered rare variants and complex polygenic risk.

Clinically, nsHTAD is a challenging subset of thoracic aortic disease. The absence of distinctive syndromic features, the limited diagnostic value of current genetic testing, and the highly variable natural history of the disease complicate risk stratification and family counseling [125]. Some individuals exhibit progressive aneurysmal enlargement before dissection, while others experience life-threatening aortic events despite only modest increases in aortic diameter [4,124]. In this context, genetic testing can substantially influence surveillance strategies, surgical decision-making, and cascade screening for relatives. Therefore, careful pre-test counseling and shared decision-making are essential before initiating a genetic evaluation [125,126]. Current practice involves the use of multigene panels including *ACTA2*, *MYH11*, *MYLK*, *PRKG1* and *LOX* to evaluate patients with suspected nsHTAD. Positive findings in these genes can refine both longitudinal surveillance and the timing of prophylactic aortic surgery. As pathogenic variants in several smooth muscle contractile genes are linked to dissections at relatively modest aortic diameters, panel-based genetic diagnosis can inform the adoption of lower surgical thresholds and more rigorous perioperative control of blood pressure and heart rate in affected individuals and at-risk relatives, even when the aorta is only mildly enlarged. Furthermore, identifying a causative variant enables cascade testing, helping to distinguish between relatives who require lifelong imaging follow-up and perioperative risk mitigation, and those who can be reassured. This improves the allocation of surveillance resources in families with nsHTAD [125,126].

### 4.1. Smooth Muscle Contractile Genes and Mechanical Homeostasis in nsHTAD

**Figure 4:**
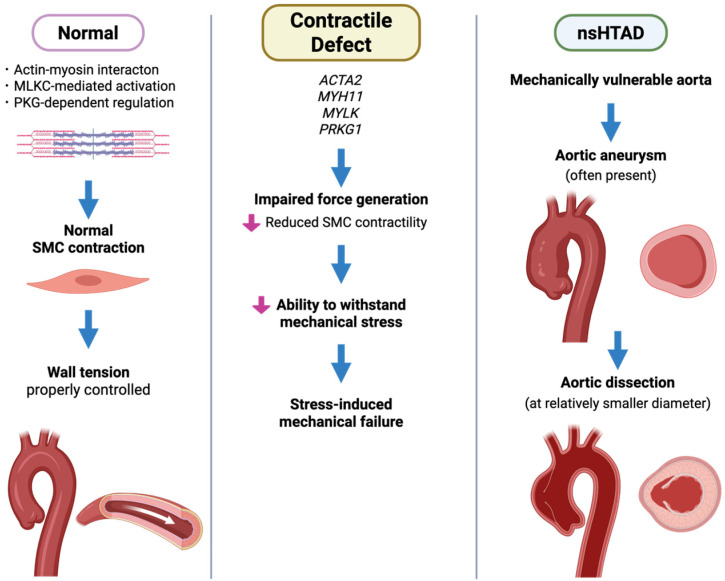
Impaired smooth muscle contractility and mechanical failure in nsHTAD; in normal aortic SMCs, the interaction between actin and myosin, as well as the regulation of this interaction by MLCK and PKG-I, maintains contractile function and appropriate control of wall tension. In nonsyndromic HTAD (nsHTAD), however, pathogenic variants in genes that encode components of the contractile apparatus (*ACTA2*, *MYH11*, *MYLK*, and *PRKG1*) impair force generation and reduce smooth muscle contractility. This diminishes the aortic wall’s ability to withstand mechanical stress, promoting stress-induced mechanical failure. Consequently, affected aortas become mechanically vulnerable and prone to aneurysm formation and dissection, which often occurs at relatively smaller diameters. Created in BioRender. Norifumi Takeda. (2026). https://app.biorender.com/illustrations/6a0ada3dd259c53aac662bc8?slideId=8806a5a9-7265-4bdf-aacf-656e1c2fe3fa (accessed on 16 April 2026).

The genes currently implicated in nsHTAD predominantly affect the contractile machinery of vascular SMCs or closely related pathways that support the mechanical integrity of the aortic wall [4,25,124]. *ACTA2*, which encodes smooth muscle α-actin, is a well-established genetic cause of HTAD among genetically resolved cases. Other genes, such as *MYH11*, *MYLK*, *PRKG1*, and *LOX*, have also been implicated in nsHTAD, highlighting additional aspects of SMC contraction, signaling, and ECM cross-linking. From a mechanobiological perspective, these genes can be grouped according to their roles in force generation, force regulation, and load transmission within the aortic wall (Figure 1 and Figure 4).

### 4.2. ACTA2 and Systemic Smooth Muscle Vasculopathy

*ACTA2*, which encodes smooth muscle α-actin, is strongly associated with nsHTAD. It accounts for approximately 15–25% of families in which a pathogenic variant is detected. Mutations in this gene impair the structure and function of the actin cytoskeleton within vascular SMCs, disrupting the generation and transmission of contractile force required to maintain aortic wall tension under pulsatile hemodynamic stress [127,128].

The vascular phenotype associated with *ACTA2* mutations extends beyond the thoracic aorta. It is increasingly recognized as a form of systemic smooth muscle vasculopathy [129]. Individuals with this condition may experience various vascular manifestations, such as early-onset ischemic stroke, occlusive cerebrovascular disease resembling Moyamoya arteriopathy, premature coronary artery disease, and peripheral arterial abnormalities. In addition, ocular findings such as iris flocculi and congenital mydriasis can serve as important clinical markers of *ACTA2*-associated disease [130,131]. Certain variants, particularly substitutions affecting Arg179, cause multisystem smooth muscle dysfunction syndrome (SMDS). This syndrome is characterized by congenital mydriasis, patent ductus arteriosus, pulmonary arterial hypertension, aortic and other arterial aneurysms, cerebrovascular arteriopathy, intestinal hypoperistalsis and malrotation, and hypotonic bladder. These symptoms highlight the systemic consequences of impaired smooth muscle contractile function [132,133,134]. Histopathological studies and clinical series demonstrate abnormal SMC morphology and medial layer disorganization across multiple vascular beds. These findings support the concept that *ACTA2* mutations impair smooth muscle function throughout the arterial system rather than producing a disease restricted to the aorta.

Another clinically important feature of *ACTA2*-associated aortopathy is that aortic dissection may occur at smaller aortic diameters than other forms of thoracic aortic aneurysm [135]. Consequently, prophylactic aortic root or ascending aortic replacement is generally recommended at lower diameter thresholds than for sporadic aortic aneurysms (Table 2). For *ACTA2*-associated aortopathy, this threshold is typically in the mid-4 cm range, particularly when there are additional high-risk features, such as a family history of early dissection or rapid aortic enlargement. For context, current practice guidelines typically recommend prophylactic surgery at approximately 5.0 cm for Marfan syndrome (or 4.5 cm if there are additional risk factors) and at around 4.0–4.5 cm for LDS. This underscores the need for gene-specific risk stratification in HTAD [5,6].

Experimental and translational studies have begun to clarify the cellular consequences of ACTA2 dysfunction. The loss or mutation of smooth muscle α-actin disrupts the assembly of actin filaments. This leads to altered cytoskeletal tension, impaired cell-matrix mechanosensing, and changes in focal adhesion and integrin signaling in vascular SMCs [136,137,138]. These alterations affect signaling pathways involved in mechanotransduction, including RhoA–ROCK signaling and actin-dependent transcriptional regulation through myocardin and serum response factor (SRF) [139,140]. These factors are central regulators of smooth muscle contractile gene expression and aortic SMC stiffness. Impaired actin dynamics are thought to promote modulation of the vascular SMC phenotype, shifting it from a contractile to a synthetic state. This synthetic state is characterized by altered ECM production, inflammation, and reduced mechanical stability of the arterial wall. Thus, *ACTA2*-associated aortopathy links cytoskeletal dysfunction to abnormal smooth muscle mechanobiology and aortic wall instability.

### 4.3. MYH11 and Smooth Muscle Contractile Failure

Pathogenic variants in *MYH11*, which encodes the smooth muscle myosin heavy chain, are an established cause of nsHTAD. MYH11 is a motor component of the actin–myosin contractile apparatus in vascular SMCs, and it is crucial for generating contractile force and maintaining arterial wall tension. Pathogenic *MYH11* variants were first identified in families with thoracic aortic aneurysms and dissections, often accompanied by patent ductus arteriosus [141]. These findings underscore the critical role of smooth muscle contractility in vascular development and postnatal arterial integrity.

*MYH11*-associated aortopathy encompasses progressive thoracic aortic aneurysms that subsequently dissect [142] as well as acute dissections arising from mildly or apparently non-dilated aortas [143] (Table 2). This suggests that MYH11 dysfunction can predispose the aortic wall to rupture even in the absence of significant aneurysmal formation. Functional and histological studies demonstrate that pathogenic *MYH11* variants impair actin–myosin interactions and smooth muscle force generation. These impairments lead to medial structural abnormalities, disorganized elastic lamellae, and reduced mechanical stability of the aortic wall [144]. A knock-in mouse model carrying the *Myh11* K1256del pathogenic variant develops aortic dissections and intramural hematomas after AngII infusion, despite only modest aortic enlargement [145,146]. These findings support the idea that additional biomechanical stressors, such as uncontrolled hypertension or abrupt increases in afterload, can cause aortic complications when the smooth muscle contractile apparatus is compromised. Furthermore, since the actin–myosin cytoskeleton is mechanically coupled to the ECM through integrin-based focal adhesions, MYH11 dysfunction may also disrupt integrin signaling and FAK activation [147,148].

### 4.4. MYLK and PRKG1: Dysregulation of Smooth Muscle Contractile Signaling

Variants in genes that regulate smooth muscle contractile signaling are another important cause of nsHTAD. Pathogenic variants in *MYLK* and *PRKG1*, which encode myosin light chain kinase (MLCK) and cGMP-dependent protein kinase I (PKG-I), respectively, highlight the critical role of intracellular signaling pathways in regulating actin–myosin contraction in vascular SMCs.

MYLK phosphorylates the regulatory light chain of myosin (MLC20), which is a critical step in the activation of actin–myosin cross-bridge cycling and smooth muscle contraction. Pathogenic *MYLK* variants were first identified in families with thoracic aortic dissection [149]. Affected individuals in these families frequently experienced acute dissections at relatively small aortic diameters, sometimes without aneurysmal dilation preceding them [150,151] (Table 2). Functional analyses have shown that *MYLK* mutations impair MLCK kinase activity [149,151]. This results in reduced phosphorylation of myosin light chains and diminished smooth muscle contractility. Together, defective activation of the actin–myosin contractile apparatus compromises the ability of the aortic wall to withstand pulsatile hemodynamic forces.

Experimental evidence further supports this concept. In murine models with reduced MYLK activity, vascular SMCs exhibit impaired contractile responses and altered cytoskeletal organization [152]. This leads to decreased mechanical resilience of the arterial wall under stress conditions. These observations reinforce the notion that defects in regulatory components of the contractile machinery, similar to structural mutations in *ACTA2* or *MYH11*, can predispose the aorta to biomechanical failure.

A distinct, yet mechanistically related, form of nsHTAD is caused by a recurrent gain-of-function mutation in *PRKG1*. The most common variant is the c.530G>A, p.(Arg177Gln) mutation [153]. *PRKG1* encodes PKG-I, a key mediator of NO–cGMP signaling that promotes smooth muscle relaxation by inhibiting actin–myosin contraction. The p.(Arg177Gln) alteration disrupts the high-affinity cGMP-binding site, constitutively activating PKG-I and decreasing the phosphorylation of the myosin regulatory light chain. This results in reduced contractile tone in vascular SMCs. Individuals with this mutation often present with early-onset thoracic aortic dissections at relatively modest aortic diameters, sometimes without significant aneurysm enlargement [153,154] (Table 2). Functional studies have demonstrated that the p.(Arg177Gln) variant results in ligand-independent activation of PKG-I and sustained phosphorylation of downstream PKG substrates [155]. These findings support a model in which excessive relaxation signaling and cytoskeletal dysregulation weaken the mechanical stability of the aortic wall. The recurrent p.(Arg177Gln) variant is the best-characterized cause of *PRKG1*-associated aortopathy. However, additional likely pathogenic missense mutations, such as p.(Gly370Ser) [156] and the recently described activating variant p.(Val219Ile) [157], expand the mutational and phenotypic spectrum of *PRKG1*-associated aortopathy.

Together, *MYLK*- and *PRKG1*-aortopathies demonstrate that disruption to contractile regulatory signaling alone, without defects to the structural components of the cytoskeleton, is sufficient to cause thoracic aortic disease. An important clinical feature of multiple mutations affecting the smooth muscle contractile apparatus is aortic dissection occurring at relatively modest aortic diameters. Prophylactic aortic surgery is often considered for smaller aortic diameters, especially in patients with a family history of early dissection.

### 4.5. LOX: Extracellular Matrix Cross-Linking and Aortic Wall Integrity

Variants in the *LOX* gene, which encodes lysyl oxidase, are a distinct genetic cause of nsHTAD that differs from mutations affecting the smooth muscle contractile apparatus in terms of mechanism. *LOX* encodes a copper-dependent enzyme that catalyzes the oxidative deamination of lysine residues in collagen and elastin. This process creates covalent cross-links that are essential for arterial tensile strength and elasticity [158]. *LOX* variants were first identified through exome sequencing in nsHTAD cohorts [159], and these conditions are characterized by fragmented elastic lamellae, disorganized ECM, and fusiform enlargement of the root or ascending aorta. While variants in *ACTA2*, *MYH11*, *MYLK*, and *PRKG1* primarily impair smooth muscle contractile function, *LOX* mutations weaken the extracellular structural scaffold that supports vascular SMCs and distributes mechanical stress within the arterial wall. *LOX* is expressed in connective tissues throughout the body. However, the dominant clinical manifestation is thoracic aortopathy, where pulsatile hemodynamic stress is greatest (Table 2).

Further support for the role of LOX in maintaining vascular stability comes from animal models. Knock-in mice that are heterozygous for human *LOX* mutations (*Lox*^M292R/+^) exhibit aortic tortuosity, fragmentation of elastic lamellae, abnormal ECM architecture, and progressive dilation of the thoracic aorta [160]. While they exhibit only subtle elastic lamellar abnormalities at baseline, they develop marked ascending aortic dilation, wall thickening, and elastin fragmentation when exposed to AngII-induced hypertension. These results suggest that a partial reduction in LOX activity is sufficient to maintain near-normal aortic architecture under physiological loads; however, it renders the aortic wall highly susceptible to increased hemodynamic stress. This emphasizes the dose-dependent requirement of LOX for vascular resilience. *LOX*-associated aortopathy is a notable example of how changes in matrix cross-linking can compromise vascular stability, predisposing the aorta to aneurysm formation and dissection.

## 5. Conclusions

HTAD is now recognized to be a disorder of aortic wall biology, rather than an isolated inherited aneurysm syndrome. Genetic studies and experimental models indicate that various mutations weaken the aortic wall by interfering with processes that influence ECM organization, TGFβ signaling, smooth muscle contractility, and biomechanical stress. Marfan syndrome, for example, is a disease driven by abnormalities in microfibrillar architecture and matrix-dependent signaling. LDS demonstrates how perturbation of TGFβ signaling can alter vascular development and stress responses. In nsHTAD, mutations affecting the smooth muscle contractile system show that impaired force generation alone can compromise aortic integrity. The current evidence suggests that the proximal thoracic aorta is a uniquely vulnerable vascular segment where developmental lineage, ECM organization, and pulsatile biomechanical stress converge. Understanding how genetic variants disrupt this integrated system may be essential for developing more precise surveillance and therapy strategies for heritable aortic disease.

Future integration of genomic variation, quantitative imaging and deep clinical phenotyping using advances in machine learning and representation learning may further improve risk prediction and disease stratification. Incorporating longitudinal data and latent disease features could allow for a more precise, personalized evaluation of aortic vulnerability that goes beyond single-variant models. In addition, emerging biomechanical approaches, including finite element analysis and hemodynamic assessment using 4D flow MRI, may provide complementary information regarding regional wall stress, flow disturbance, and vascular stiffness. Studies in Marfan syndrome have already shown that 4D flow MRI-derived alterations in wall shear stress and flow patterns can be detected even before overt aortic dilatation and may relate to subsequent regional enlargement of the proximal and descending thoracic aorta [161,162]. These approaches may help identify biomechanically vulnerable regions of the aorta that are not fully captured by aortic diameter alone. Integration of genetic, imaging, and biomechanical data could therefore improve individualized risk assessment and refine the timing of surveillance and prophylactic intervention in HTAD.

## Figures and Tables

**Table 1:** Syndromic TGFβ-pathway aortopathies.

Gene	OMIM (Gene/Disease)	Disease	Molecular Role	Vascular Phenotype	Systemic Features	Clinical Considerations
*FBN1*	* 134797/# 154700	Marfan syndrome	Structural component of extracellular microfibrils	Predominant aortic root dilation with risk of type A dissection	Tall stature, long extremities, chest wall and spine deformities, pneumothorax, and ectopia lentis are characteristic.	Variant class (haploinsufficiency vs. dominant-negative) has been associated with differences in severity and progression in several studies.
*TGFBR1*	* 190181/# 609192	LDS, type 1	TGFβ receptor	Widespread arterial aneurysms and dissections with tortuosity across multiple vascular beds.	Craniofacial, cutaneous, and skeletal features including hypertelorism, bifid uvula or cleft palate, and pectus deformity may be present.	Some genotype-related variability and possible sex differences in clinical severity have been suggested, but findings remain inconsistent across studies.
*TGFBR2*	* 190182/# 610168	LDS, type 2	TGFβ receptor	Diffuse arterial aneurysms and dissections with marked tortuosity. Aortic events may occur at smaller diameters in some patients.	Craniofacial, cutaneous, and skeletal features typical of LDS, such as hypertelorism and bifid uvula or cleft palate, may be present but are variable.	Clinical course can be relatively aggressive in some patients, and current guidelines consider smaller body size, extra-aortic features, family history of aortic dissection, and rapid aortic growth as potential risk modifiers when planning prophylactic surgery.
*SMAD2*	* 601366/# 618355	LDS, type 6	Intracellular mediator of TGFβ signaling	Aortic and other arterial aneurysms and dissections with arterial tortuosity have been reported, often involving the thoracic aorta and additional arterial beds.	Craniofacial, cutaneous, and skeletal features consistent with LDS, including hypertelorism and cleft or bifid uvula or palate, may be present.	Available data suggest an LDS phenotype, but reported numbers remain small and genotype–phenotype correlations for *SMAD2* are still being defined.
*SMAD3*	* 603109/# 613795	LDS, type 3 (AOS)	Intracellular mediator of TGFβ signaling	Thoracic aortic aneurysms and aneurysms in other arterial beds with variable tortuosity have been reported.	Early-onset osteoarthritis, degenerative disc disease, and mild craniofacial or skeletal features may be present.	Represents a phenotype combining vascular and osteo-articular involvement; penetrance of systemic features is relatively high.
*TGFB2*/*TGFB3*	*TGFB2* * 190220/# 614816; *TGFB3* * 190230/# 615582	LDS, type 4/type 5	TGFβ ligands	Arterial aneurysms involving thoracic and extra-thoracic vessels, generally with milder or later onset compared with receptor variants.	Systemic features are often milder or subtle, and classic craniofacial or skeletal findings may be absent.	Marked intra- and interfamilial variability; genotype–phenotype correlations remain incompletely defined.
*PMEPA1*	* 606564/(LDS-like aortopathy; no established OMIM disease entry)	LDS-like aortopathy	Negative regulator of TGFβ signaling	Aortic aneurysm and related aortopathy have been reported in a limited number of families.	Extra-vascular features resembling connective tissue disorders have been variably described and are not yet well characterized.	Evidence is based on small cohorts; clinical spectrum and management implications remain to be established.

LDS indicates Loeys–Dietz syndrome; AOS, aneurysm-osteoarthritis syndrome; *, gene entry; and #, phenotype entry. Clinical and mechanistic features summarized from previously published studies and reviews [2,3,4,5,6,7].

**Table 2:** Nonsyndromic HTAD associated with smooth muscle dysfunction and ECM abnormalities.

Gene	OMIM (Gene/Disease)	Molecular Role	Aortic Phenotype	Dissection Characteristics	Systemic Features
*ACTA2*	* 102620/# 611788	Smooth muscle contractile protein (α-actin)	Predominantly aortic root and ascending aortic aneurysm has been reported, and additional arterial involvement may occur in some individuals.	Thoracic aortic dissection can occur, occasionally at relatively modest diameters or younger ages.	Extra-aortic features are often subtle and may include iris anomalies such as iris flocculi and congenital mydriasis, and cerebrovascular or coronary involvement has been observed in a subset of patients.
*MYH11*	* 160745/# 132900	Smooth muscle contractile protein (myosin heavy chain)	Familial thoracic aortic aneurysm, typically involving the ascending aorta, has been described.	Aortic dissection has been reported, sometimes in the context of PDA or altered hemodynamics.	Systemic features are generally limited, and childhood PDA may serve as a clinical clue in some families.
*MYLK*	* 600922/# 613780	Regulator of smooth muscle contraction (MLCK)	Aortic dilation may be mild or absent, with marked variability between affected individuals.	Acute dissection or rupture can occur over a wide age range, even without substantial preceding dilation.	Systemic features are usually absent or minimal, and many individuals appear to be nonsyndromic.
*PRKG1*	* 176894/# 615436	cGMP-dependent signaling kinase in vascular smooth muscle	Relatively mild thoracic aortic disease with proximal aortic enlargement has been reported in several families.	Early-onset dissections, sometimes aggressive and including events at small diameters, have been described.	Extra-aortic features are not clearly defined and generally appear minimal.
*LOX*	* 153455/# 617168	ECM cross-linking enzyme for elastin and collagen	Familial aortic root or ascending aortic aneurysm with fusiform enlargement has been observed.	Ascending aortic dissection has been reported, and some cases suggest that events may occur before extreme aortic enlargement.	Systemic features are generally rare, and no consistent syndromic pattern has been identified.

PDA indicates patent ductus arteriosus; MLCK, myosin light chain kinase; ECM, extracellular matrix; *, gene entry; and #, phenotype entry.

## Data Availability

No new data were created or analyzed in this study. Data sharing is not applicable to this article.

## Abbreviations

The following abbreviations are used in this manuscript:

HTAD	Heritable thoracic aortic diseases
nsHTAD	Nonsyndromic Heritable thoracic aortic diseases
LDS	Loeys–Dietz syndrome
ECM	Extracellular matrix
TGFβ	Transforming growth factor-β
SMC	Smooth muscle cell
vEDS	vascular Ehlers–Danlos syndrome
CNC	Cardiac neural crest
SHF	Second heart field
AngII	Angiotensin II
MMP	Matrix metalloproteinase
ARB	AngII type 1 receptor blocker
AT1R	AngII type 1 receptor
AT2R	AngII type 2 receptor
NO	Nitric oxide
NOX4	NADPH oxidase 4
GWAS	Genome-wide association study
MLCK	Myosin light chain kinase
PKG-I	cGMP-dependent protein kinase I
AOS	Aneurysm-osteoarthritis syndrome
PDA	Patent ductus arteriosus
